# COVID-19 associates with semen inflammation and sperm quality impairment that reverses in the short term after disease recovery

**DOI:** 10.3389/fphys.2023.1220048

**Published:** 2023-07-11

**Authors:** María Sol Martinez, Fernando Nicolás Ferreyra, Daniela Andrea Paira, Virginia Elena Rivero, José Javier Olmedo, Andrea Daniela Tissera, Rosa Isabel Molina, Rubén Darío Motrich

**Affiliations:** ^1^ CIBICI-CONICET, Facultad de Ciencias Químicas, Universidad Nacional de Córdoba, Córdoba, Argentina; ^2^ Fundación Urológica Córdoba para la Docencia e Investigación Médica (FUCDIM), Córdoba, Argentina; ^3^ Laboratorio de Andrología y Reproducción (LAR), Córdoba, Argentina

**Keywords:** COVID-19, SARS-CoV-2, sperm quality, inflammation, male infertility, cytokine

## Abstract

**Introduction:** COVID-19 exerts deleterious effects on the respiratory, cardiovascular, gastrointestinal, and central nervous systems, causing more severe disease in men than in women. However, cumulative reported data about the putative consequences on the male reproductive tract and fertility are controversial. Furthermore, the long-term effects of SARS-CoV-2 infection are still uncertain.

**Methods:** In this study, we prospectively evaluated levels of inflammatory cytokines and leukocytes in semen and sperm quality parameters in a cohort of 231 reproductive-aged male patients, unvaccinated, who had recovered from mild or severe COVID-19 and in 62 healthy control individuals. Sperm quality was assessed early (less than 3 months) and long (more than 3 and up to 6 months) after having COVID-19. Interestingly, and unlike most reported studies, available extensive background and baseline data on patients’ sperm quality allowed performing a more accurate analysis of COVID-19 effects on sperm quality.

**Results:** Significantly higher levels of IL-1β, TNF and IFNγ were detected in semen from patients recently recovered from mild and/or severe COVID-19 with respect to control individuals indicating semen inflammation. Moreover, patients recovered from mild and/or severe COVID-19 showed significantly reduced semen volume, lower total sperm counts, and impaired sperm motility and viability. Interestingly, all observed alterations returned to baseline values after 3 or more months after disease recovery.

**Discussion:** These results indicate that COVID-19 associates with semen inflammation and impaired semen quality early after disease. However, long COVID-19 seems not to include long-term detrimental consequences on male fertility potential since the observed alterations were reversible after 1-2 spermatogenesis cycles. These data constitute compelling evidence allowing a better understanding of COVID-19 associated sequelae, fundamental for semen collection in assisted reproduction.

## 1 Introduction

The SARS-CoV-2 coronavirus disease (COVID-19) outbreak originated in China in December 2019 and rapidly spread worldwide causing a pandemic. Since then, more than seven hundred million cases of COVID-19 and almost 7 million deaths have been reported up to May 2023 (OurWorldinData, 2023). Although it affects both, women and men, COVID-19 can disproportionately affect and cause more harm and a higher mortality rate to men (Zsichla and Muller, 2023). Androgens have been implicated as the underlying cause for more severe disease, since testosterone has been shown to correlate with higher SARS-CoV-2 infection rates, the induction of weaker immune responses, and increased risk of thromboembolic events (Pivonello et al., 2021). The clinical presentation of COVID-19 is variable ranging from asymptomatic infection to fatal disease, with still unknown long-term consequences. In fact, there is increasing awareness that many patients continue to have heterogeneous, long term post-acute sequelae of COVID-19 or long COVID disease (Reese et al., 2023). Although still unclear, long COVID generally refers to a multisystemic condition comprising ongoing, relapsing or new symptoms including fatigue, cough, headache, palpitations, arthralgia, diarrhea, memory or cognitive dysfunctions, irregular menstruation, and erectile dysfunction, or other health consequences that last or occur four or more weeks after the initial SARS-CoV-2 infection (Davis et al., 2023; Reese et al., 2023). Although the current understanding of the natural history of long COVID-19 is still incomplete, it has been shown to affect individuals of all ages, having had symptomatic or asymptomatic acute infections, mostly with mild and non-hospitalized disease and in patients aged 36–50 years (Ballering et al., 2022). Estimates on currently reported data indicate that at least 65 million people suffer from long COVID-19 worldwide (Ballering et al., 2022; Reese et al., 2023). However, this number is likely much higher due to the high number of subclinical and/or undocumented cases.

In that regard, and although still under debate, cumulative reported evidence indicates that SARS-CoV-2 infection impairs and it may actually have detrimental consequences on male fertility. In fact, it has been shown that COVID-19 negatively correlates with sperm quality parameters such as volume, concentration, motility, and viability, these changes often correlating with disease severity (Gacci et al., 2021; Guo et al., 2021). Interestingly, a systematic review and meta-analysis has shown that mild/asymptomatic COVID-19 also has detrimental effects on sperm quality by decreasing sperm concentration, total sperm count, progressive and total sperm motility, and normal sperm morphology (Che et al., 2022). However, the high heterogeneity among reported studies, based on technical differences, not uniform diagnostic criteria, different research centers and hospitals, and, most importantly, the absence of baseline semen parameters data before the infection, may cause certain deviations in the analysis outcome of COVID-19 effects on sperm quality. Furthermore, there are very scarce reported data assessing the precise relationship between the onset of the infection with baseline data and any later changes in sperm quality. On the other hand, the lack of long follow-up cohort studies poses many questions about the putative serious and long-lasting effects of SARS-CoV-2 infection on sperm quality and male fertility potential (Che et al., 2022; Davis et al., 2023).

For these reasons, we herein performed a prospective analysis of semen inflammation, i.e., levels of inflammatory cytokines and leukocytes in semen, and sperm quality parameters in a cohort of reproductive-aged male patients recovered from mild or severe COVID-19 and in healthy control individuals. In addition, sperm quality was assessed pre-COVID-19, early (from diagnosis to 3 months post-infection) and long (more than 3 and up to 6 months post-infection) after COVID-19. Noteworthy, extensive background and baseline data on patients’ sperm quality was available, which allowed performing accurate analysis of COVID-19 effects on sperm quality.

## 2 Materials and methods

### 2.1 Study design, subjects and samples

This prospective cross-sectional study included two hundred ninety three (*n* = 293) reproductive-aged male individuals attending a joint Academic, Urology and Andrology clinic between June 2020 and November 2021. The subject population included 231 patients, unvaccinated, who had mild (*n* = 199, aged 20–47 years) or severe (*n* = 32, aged 30–55 years) COVID-19 and 62 healthy control individuals (aged 22–49 years). The inclusion criteria for patients with COVID-19 were as follows: 1) males aged 20–55 years, 2) unvaccinated, and 3) having recently had laboratory confirmed (PCR) mild or severe COVID-19. The distinction between patients having mild or severe COVID-19 was made based on the fact of having symptomatic COVID that required hospitalization irrespective of the need of mechanical ventilation (severe) or not (mild). The inclusion criteria for controls were: 1) males aged 20–55 years, 2) unvaccinated, and 3) no history of COVID-19. The exclusion criteria for patients and controls were as follows: 1) vasectomy, 2) history of urogenital surgery, 3) azoospermia, 4) body mass index (BMI) higher than 30.0 kg/m^2^, 5) diagnosis of grade 3–4 varicocele, 6) toxic/pollutant exposure, 7) smoking (more than 10 cigarettes per day), 8) alcohol (more than five cups per day) or marijuana consumption, and/or 9) recent genitourinary tract infection/inflammation (during the precedent 12 weeks). After being advised of the purpose and potential risks of the study, all participants provided written informed consent to participate in the study and share their own anonymous information. All enrolled patients and control individuals underwent the analysis performed in the study. The study was approved by the Institutional Ethics Committee from the National University of Cordoba (RePIS #3512) and conducted in accordance with the Code of Ethics of the World Medical Association (Declaration of Helsinki) standards and the Argentinian legislation for protection of personal data (Law 25326).

### 2.2 Semen collection

Semen samples were collected by masturbation into a sterile container after 3–5 days of sexual abstinence. The samples were liquefied at 37°C for about 20 min in an incubator before analysis. Semen samples were separated into two aliquots, one to perform semen analysis and the remaining one to obtain seminal plasma to assess cytokine levels. Seminal plasma was obtained after centrifuging semen aliquots at 400 g for 15 min. The recovered seminal plasma was immediately examined under a microscope, ensuring the absence of cells, and then stored at −80°C until use.

### 2.3 Semen analysis

Semen analysis was performed according to the World Health Organization Semen Analysis Manual (WHO, 2010). Semen analysis was performed at least twice in each individual. Sperm concentration, total and progressive motility were assessed within 1 h after ejaculation. Sperm viability was analyzed using eosin Y 0.5% (Sigma-Aldrich, St. Louis, MO, United States) staining. Sperm morphology was evaluated by the Papanicolaou technique and according to Kruger’s strict criteria and WHO’s criteria. The concentration of round cells was evaluated using the Makler counting chamber (Sefi-Medical Instrument, Haifa, Israel) and peroxidase-positive cells (as a mean of leukocytes) were quantified among round cells using a previously described cytochemical assay (Politch et al., 1993). Anti-sperm antibodies (IgG and IgA) were evaluated by the direct SpermMar test (FertiPro, Belgium) and considered positive when sperm showing bound latex particles were ≥50%. Routine sperm parameters were assessed in at least 200 spermatozoa per sample by two operators, rendering a total of 400 scored spermatozoa.

### 2.4 Quantification of cytokines in semen

IL-8, TNF, IL-1β, IL-6, IFNγ, IL-10 and IL-17A concentrations in seminal plasma were analyzed using ELISA specific kits according to the manufacturer’s instructions. IL-8, TNF and IL-6 were quantitated by the respective BD OptEIA™ kits (BD Biosciences Pharmingen, San Diego, CA, United States). IL-17A, IFNγ, IL-10 and IL-1β were assayed by the respective ELISA Ready-SET-Go kits (eBioscience, San Diego, CA, United States). Samples were analyzed at least in triplicates and the results were expressed as pg/mL. In the case of IL-8 quantitation, seminal plasma samples were assayed diluted 1/20.

### 2.5 Statistical analysis

Statistical analysis was performed using the GraphPad Prism software, version 9.5 (GraphPad Inc., La Jolla, CA, United States). Data distribution was assessed by the Shapiro-Wilk test. Data were analyzed using the Kruskal-Wallis non-parametric test and the Friedman test (repeated measures in time) as appropriate. Differences were considered statistically significant when *p* < 0.05.

## 3 Results

### 3.1 COVID-19 associates with semen inflammation in recently recovered patients

**FIGURE 1:**
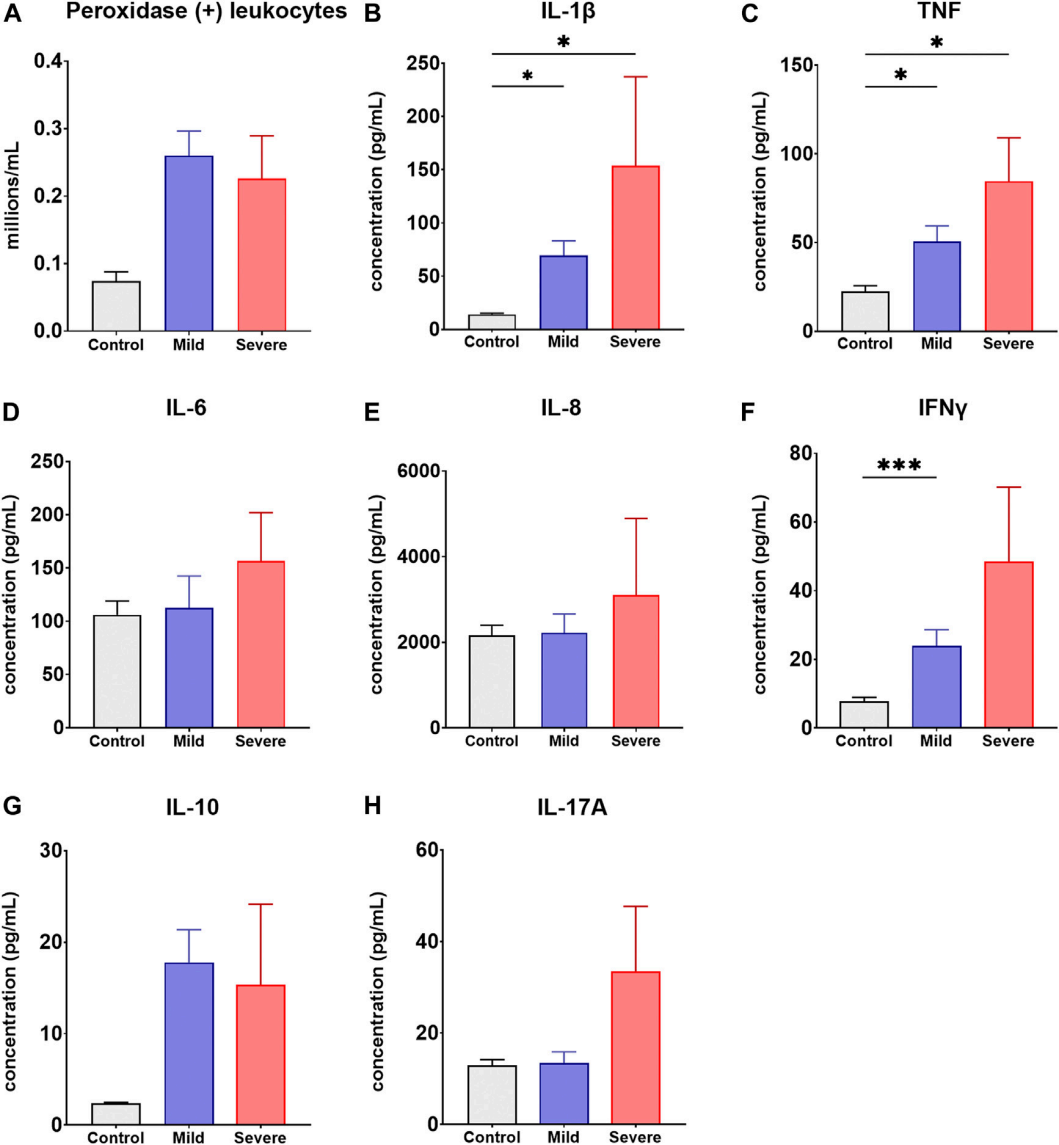
Inflammatory biomarkers in semen from recently recovered COVID-19 patients and healthy control individuals. Concentrations of peroxidase-positive leukocytes **(A)**, IL-1β **(B)**, TNF **(C)**, IL-6 **(D)**, IL-8 **(E)**, INFγ **(F)**, IL-10 **(G)** and IL-17A **(H)** in semen specimens from patients recently recovered (up to 3 months after infection confirmation) from either mild (*n* = 199) or severe (*n* = 32) COVID-19 and healthy control individuals (*n* = 62). Samples were assessed at least in triplicate. Data are shown as mean ± SEM. Kruskal-Wallis non-parametric test was used; *p < 0.05, and ***p < 0.001.

Based on inclusion/exclusion criteria, 231 unvaccinated patients recently recovered (up to 3 months after infection confirmation) from either mild (*n* = 199, median age = 36) or severe (*n* = 32, median age = 39) COVID-19 and 62 unvaccinated healthy control individuals (*n* = 62, median age = 36) were enrolled in the study. Regarding the infection, no personalized analysis of the particular SARS-CoV-2 viral strain which every patients was infected with was performed. Nevertheless, available epidemiological data from our country indicate that the viral variants circulating in the period when patients under study got the infection were mainly the Gamma (lineage P.1, derived from lineage B.1.1.28; detected in 60% of cases) and Lambda (lineage C.37, derived from B.1.1.1; detected in 25% of cases) variants (Argentina, 2021). To assess if COVID-19 associates with semen inflammation, levels of different inflammatory cytokines (i.e., IL-1β, TNF, IL-6, IL-8, IFNγ, IL-10 and IL-17A) and peroxidase-positive leukocytes (i.e., neutrophils and activated macrophages) in semen from patients and controls were analyzed. As shown in Figure 1A, no significant differences were observed in the semen counts of peroxidase-positive leukocytes between patients recently recovered from COVID-19, either mild or severe, and controls. However, significantly higher levels of IL-1β, TNF and IFNγ were found in semen from patients recovered from mild and/or severe COVID-19 with respect to control individuals (*p* < 0.05, *p* < 0.05 and *p* < 0.001, respectively; Figures 1B, C, F). Conversely, no significant differences in the semen levels of IL-6, IL-8, IL-10 and IL-17A were found between patients and controls (*p* > 0.05, Figures 1D, E, G, H). These results indicate that mild and/or severe COVID-19 associates with semen inflammation up to 3 months after disease diagnosis.

### 3.2 COVID-19 impairs sperm quality in recently recovered patients

**FIGURE 2:**
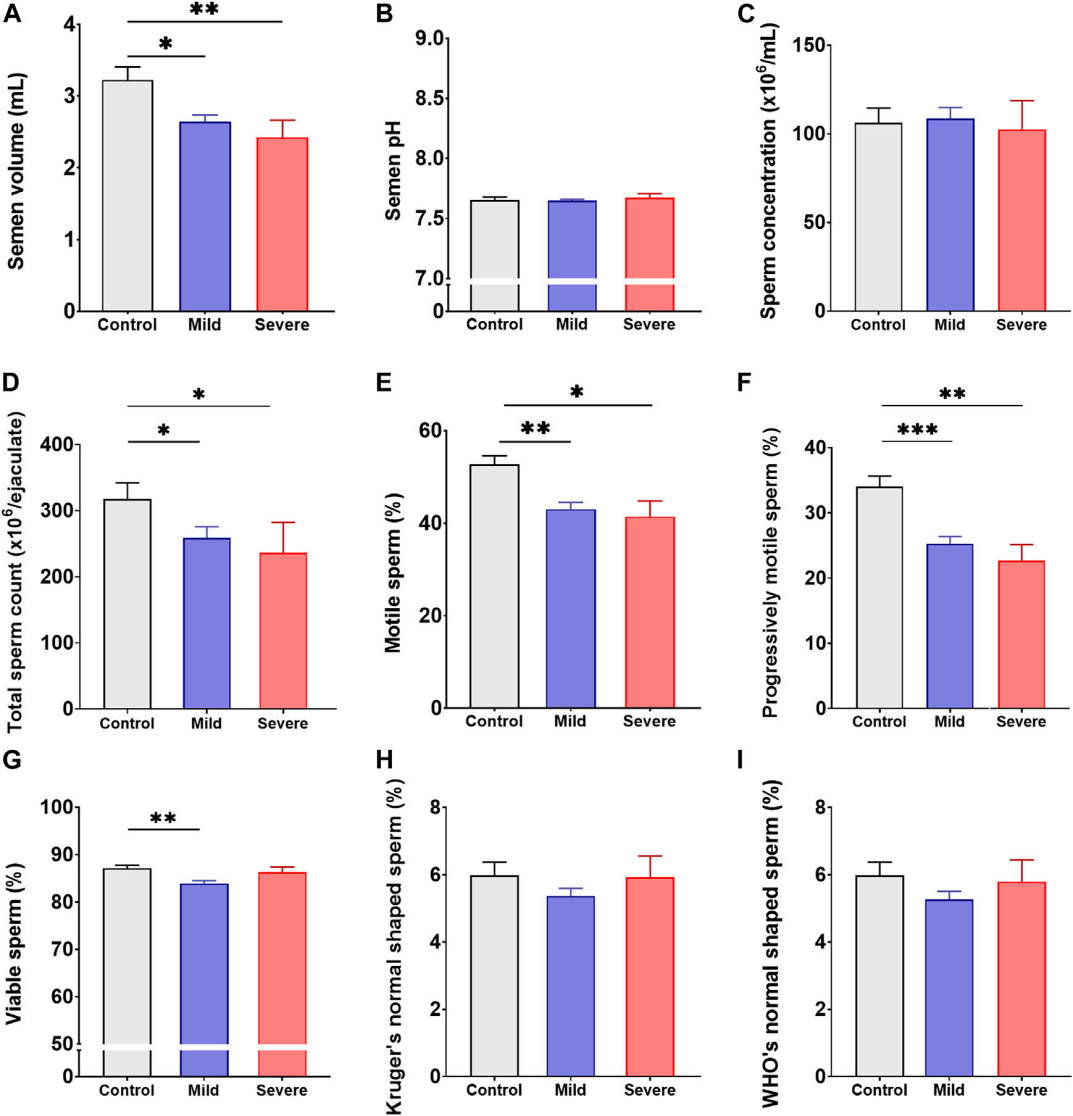
Sperm quality from recently recovered COVID-19 patients and healthy control individuals. Assessment of semen volume **(A)**, pH **(B)**, sperm concentration **(C)**, total sperm count **(D)**, sperm total motility **(E)**, sperm progressive motility **(F)**, sperm viability **(G)**, and normal sperm morphology according to the Kruger’s **(H)** or the WHO’s **(I)** criteria in semen specimens from patients recently recovered (up to 3 months after infection confirmation) from either mild (*n* = 199) or severe (*n* = 32) COVID-19 and healthy control individuals (*n* = 62). Semen analysis was performed at least twice in each individual. Data are shown as mean ± SEM. Kruskal-Wallis non-parametric test was used; *p < 0.05, **p < 0.01 and ***p < 0.001.

To assess whether COVID-19 would impair male fertility potential, sperm quality parameters from patients recently recovered (up to 3 months after infection confirmation) from either mild or severe COVID-19 and healthy controls were analyzed. Remarkably, abnormal sperm quality [i.e., when one or more semen quality parameter showed values falling without the WHO 2010 reference values (WHO, 2010)] were observed in a higher proportion of patients recently recovered from either mild (87.4%) or severe (71.9%) COVID-19 than in control individuals (19.4%) (Table 1). When assessing different sperm quality parameters, patients recently recovered from mild and/or severe COVID-19 showed significantly reduced semen volume (*p* < 0.05 and *p* < 0.01), lower total sperm counts (*p* < 0.05 and *p* < 0.05), impaired total (*p* < 0.01 and *p* < 0.05) and progressive sperm motility (*p* < 0.001 and *p* < 0.01), and reduced viability (*p* < 0.01) (Figures 2A, D–G; Supplementary Table S1). On the contrary, no significant differences were found in semen pH, sperm concentration and morphology between patients and controls (*p* > 0.05, Figures 2B, C, H, I; Supplementary Table S1). Noteworthy, no anti-sperm IgG or IgA antibodies were found in any patient or control individual under study (Supplementary Table S1). These results indicate that COVID-19 reduces sperm quality at least up to 3 months after disease diagnosis and thus might impair male fertility potential.

**TABLE 1:** Frequency of semen alterations in COVID-19 patients and control individuals.

Subjects	Normospermia (*n*, %)	Abnormal semen quality (*n*, %)
Controls (*n* = 62)	50 (80.60%)	12 (19.40%)
Mild COVID-19 patients (*n* = 199)	25 (12.60%)	174 (87.40%)
Severe COVID-19 patients (*n* = 32)	9 (28.10%)	23 (71.90%)

### 3.3 COVID-19 associated sperm quality impairments are reversible

**FIGURE 3:**
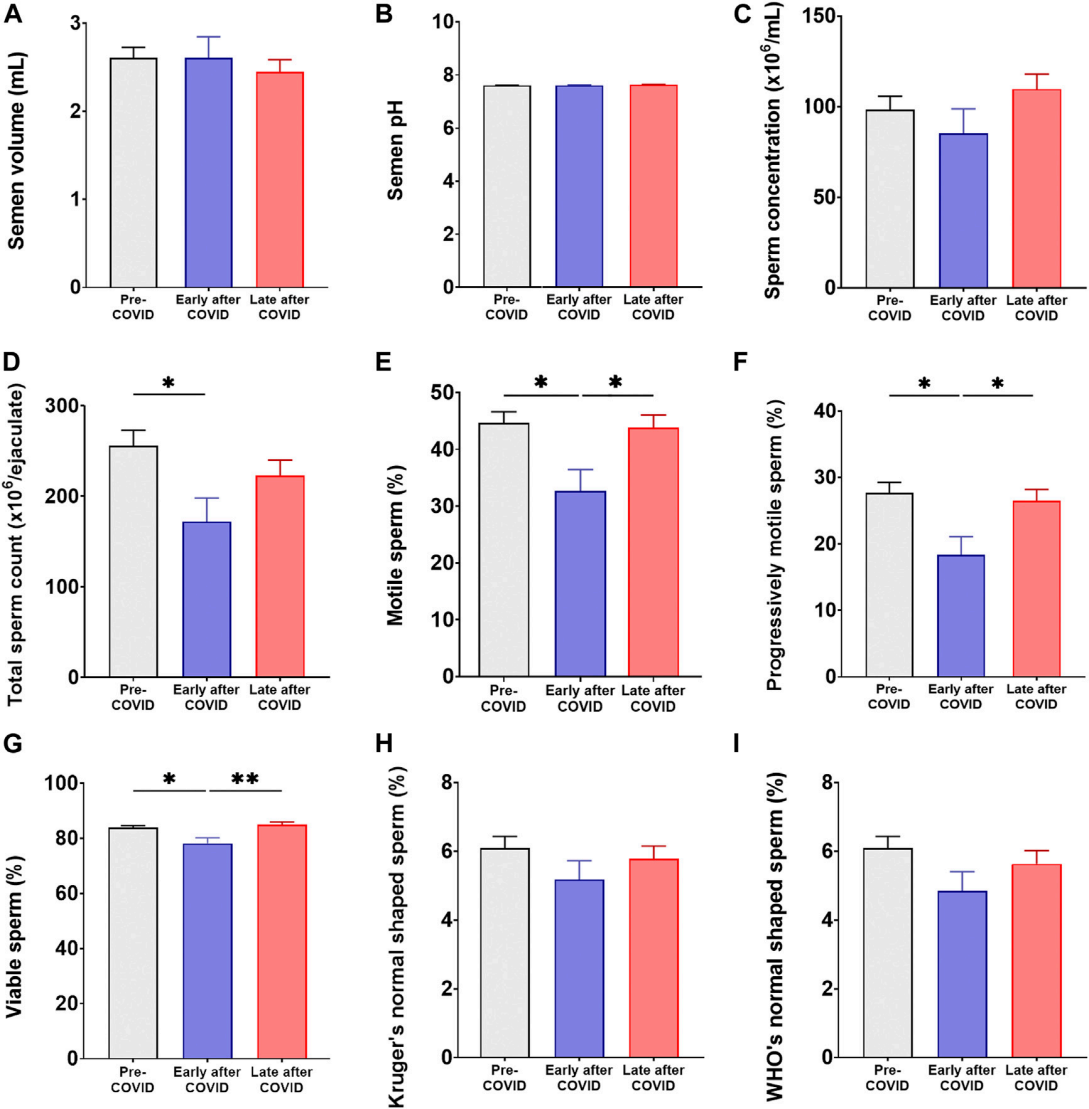
Longitudinal analysis of sperm quality in COVID-19 patients before the infection and early and long after disease. Assessment of semen volume **(A)**, pH **(B)**, sperm concentration **(C)**, total sperm count **(D)**, sperm total **(E)** and progressive motility **(F)**, sperm viability **(G)**, and normal sperm morphology according to the Kruger’s **(H)** or the WHO’s **(I)** criteria in semen specimens from patients before SARS-CoV-2 infection and early and long after COVID-19, irrespective of disease severity (*n* = 231). Semen analysis was performed at least twice in each individual. Data are shown as mean ± SEM. Friedman test (repeated measures in time) was used; *p < 0.05, and **p < 0.01.

As conflicting data about COVID-19 and sperm quality has been reported (Gacci et al., 2021; Gupta et al., 2021; Patel et al., 2021; Aksak et al., 2022; Che et al., 2022; Corona et al., 2022; Nassau et al., 2022; Tufvesson et al., 2022; Paoli et al., 2023), the potential reversibility of the observed impaired sperm quality after more than 1 spermatogenesis cycle was analyzed. To do that, sperm quality was analyzed comparing available baseline data from every patient before the infection and early (less than 3 months) and late (more than 3 and up to 6 months) after COVID-19 diagnosis, irrespective of disease severity. In agreement with results presented above, data shown in Figure 3 indicate that COVID-19 does impair sperm quality by reducing total sperm count (*p* < 0.05), sperm total and progressive motility (*p* < 0.05 and *p* < 0.05, respectively), and viability (*p* < 0.05) early after disease diagnosis with respect to baseline data before the infection (Figures 3D–G; Supplementary Table S2). However, no such differences were found when analyzing sperm quality parameters after a period of more than 3 months after disease diagnosis (*p* > 0.05, Figure 3; Supplementary Table S2). These results indicate that although COVID-19 does impair sperm quality, the observed alterations are reversible and gradually return to baseline levels after 3 months from disease recovery.

## 4 Discussion

Herein, we are reporting data of a cross-sectional study assessing semen inflammation and sperm quality in reproductive-aged male individuals recovered from mild or severe COVID-19. Interestingly, we prospectively assessed sperm quality before and early and long after SARS-CoV-2 infection. Our results indicate that patients recently recovered from mild and/or severe COVID-19 show semen inflammation as evidenced by elevated seminal plasma levels of IL-1β, TNF and IFNγ with respect to control individuals. Associated to semen inflammation, most patients recovered from mild and/or severe COVID-19 showed impaired semen quality due to significantly reduced semen volume, lower total sperm counts, and impaired sperm motility and viability. Interestingly, when analyzing sperm quality before and after disease, results show that although COVID-19 was associated with reduced sperm quality early after disease diagnosis, all observed alterations returned to baseline values after three or more months. Altogether, these data indicate that COVID-19 associates with semen inflammation and impaired sperm quality early after disease. However, long COVID-19 seems not to include detrimental consequences on male fertility potential since the observed alterations were reversible after 3 months from disease recovery.

Epidemiological data have showed men to be at higher risk of COVID-19 susceptibility and increased mortality than women (Haitao et al., 2020). Although much has been known about COVID-19 physiopathology since the SARS-CoV-2 pandemic emerged more than 3 years ago, there are several aspects that still remain unknown, especially those related to long term post-acute sequelae of COVID-19 (Maglietta et al., 2022; Davis et al., 2023; Reese et al., 2023). Remarkably, we already know that COVID-19 may have consequences on the respiratory, cardiovascular, gastrointestinal and central nervous systems; however, there is still controversy about the putative effects on the reproductive tract and fertility since conflicting data has been reported (Corona et al., 2022; Nassau et al., 2022; Dai et al., 2023). Some available evidence have shown that sperm quality is not significantly affected by the infection or the associated-disease (Gupta et al., 2021; Ruan et al., 2021; Temiz et al., 2021; Hu et al., 2022; Paoli et al., 2023). On the contrary, several other reported studies indicate that SARS-CoV-2 infection exerts adverse effects on sperm quality and male fertility potential. It has been shown that clinically severe or mild and even asymptomatic SARS-CoV-2 infection was followed by a decline in semen volume and sperm concentration, total sperm counts, motility and/or viability (Holtmann et al., 2020; Erbay et al., 2021; Gacci et al., 2021; Koc and Keseroglu, 2021; Pazir et al., 2021; Aksak et al., 2022; Ertas et al., 2022). Nevertheless, if such disease-associated deleterious effects on sperm quality are reversible or long lasting is still uncertain since scarce data has been reported in that regard (Guo et al., 2021; Donders et al., 2022). Thus, reported evidence are heterogeneous, controversial and, importantly, they mostly lack individual status data before the infection and in the early as well as late periods after disease, which would allow comprehensively data interpretation and drawing proper conclusions (Che et al., 2022; Dos Santos et al., 2022; Tufvesson et al., 2022; Xie et al., 2022). Therefore, the short and long-term effects of SARS-CoV-2 infection on sperm quality and male fertility remain to be unveiled (Davis et al., 2023; Reese et al., 2023). Consequently, there is an urgent need for compelling data to accurately understand the pathophysiology and consequences of SARS-CoV-2 infection on semen inflammation, sperm quality and male fertility potential. Herein, we provide evidence from a longitudinal study assessing semen inflammation and sperm quality in a considerable number of COVID-19 patients and healthy control individuals. Our data are in agreement with and support reported evidence indicating that COVID-19 exerts deleterious effects on sperm quality early after disease, and thus might impair male fertility potential. Remarkably, and unlike most reported studies, we had extensive sperm quality baseline data from patients before SARS-CoV-2 infection, and early and long after COVID-19, which allowed distinguishing infection consequent from pre-existing conditions. Interestingly, and supporting the very scarce reported evidence with baseline data (Gharagozloo et al., 2022; Seckin et al., 2022) and with long follow-up of patients (Guo et al., 2021; Donders et al., 2022; Gharagozloo et al., 2022; Hu et al., 2022; Seckin et al., 2022), our results indicate that the observed decline of sperm quality after symptomatic SARS-CoV-2 infection progressively recovered after 3 months from disease recovery.

Different possible direct and indirect effects of the infection and associated disease (COVID-19) could underlie the observed sperm alterations and impaired spermatogenesis (Dai et al., 2023). First, the putative detrimental impact of SARS-CoV-2 infection on testicular function. Indeed, testicular histopathological alterations including germ cell destruction, thinning of seminiferous tubules, increased numbers of apoptotic cells and a decrease in the number of spermatozoa in the tubules, Sertoli cell swelling and detachment, loss of Leydig cells, leukocytic infiltration and IgG deposits have already been described in COVID-19 patients (Li et al., 2020; Yang et al., 2020). Moreover, Chet et al*.* identified signs compatible with acute orchitis or epididymo-orchitis in 22.5% hospitalized COVID-19 patients (Chen et al., 2021). Moreover, authors observed that men with severe COVID-19 had a significantly higher possibility of epididymo-orchitis compared to the non-severe COVID-19 patients (Chen et al., 2021). SARS-CoV-2 uses the angiotensin-converting enzyme 2 (ACE2) receptor and the cell protease type II transmembrane serine protease (TMPRSS2) to bind to the cell and for virus-cell fusion (Hoffmann et al., 2020). As high expression levels of ACE2 and TMPRSS2 have been described in spermatogonial stem cells, peritubular myoid cells, Leydig and Sertoli cells (Fu et al., 2020; Wang and Xu, 2020; Qi et al., 2021), the testis has been proposed as a highly susceptible target organ for SARS-CoV-2 infection. However, that is controversial since ACE2 and TMPRSS2 are not co-expressed on a cellular level in testicular tissue (Baughn et al., 2020; Stanley et al., 2020; Borges et al., 2021), a fact that suggest the testis would not be susceptible to infection. In agreement, the current main consensus is that the chance of detecting the virus in semen is negligible since the vast majority of the extensively performed studies showed negative results for the presence of viral RNA or proteins (Paoli et al., 2021; Ata et al., 2022; Corona et al., 2022). Moreover, there is no current evidence that SARS-CoV-2 can be sexually transmitted (Ata et al., 2022; Nassau et al., 2022). However, it has been shown that SARS-CoV-2 can use other cell receptors to invade the cell such as CD147 or Basigin (BSG) with cysteine protease cathepsin L (CTSL) as the protease (Ulrich and Pillat, 2020; Vilella et al., 2021; Dai et al., 2023). As BSG and CSTL co-expression has been reported in early and late primary spermatocytes (Stanley et al., 2020), the possibility of testicular infection and spermatogenesis impairment by SARS-CoV-2 cannot be ruled out. Nevertheless, recently reported data indicates that testicular injury associated with SARS-CoV-2 infection is likely an indirect effect of exposure to systemic inflammation and/or SARS-CoV-2 antigens since the virus does not productively infect any testicular cell type (Giannakopoulos et al., 2023).

Second, spermatogenesis impairment may be caused by the infection-associated inflammation and oxidative stress (Bachir and Jarvi, 2014; Farsimadan and Motamedifar, 2020). In fact, we detected semen inflammation in patients recently recovered from mild and/or severe COVID-19 as shown by increased levels of IL-1β, TNF and IFNγ in seminal plasma. Our findings are in agreement with previously reported data showing increased levels of inflammatory cytokines, such as IL-1β, TNF and IFNγ, and oxidative stress together with impaired spermatogenesis in men recovering from COVID-19 (Li et al., 2020; Gacci et al., 2021; Hajizadeh Maleki and Tartibian, 2021; Morselli et al., 2022; Shcherbitskaia et al., 2022). Interestingly, seminal levels of IL-1β and TNF have been shown to negatively correlate with total sperm count and concentration in SARS-CoV-2 infected patients, being the higher levels of IL-1β detected in the group of crypto-azoospermic patients (Morselli et al., 2022). Elevated levels of inflammatory cytokines in semen could be resultant of testicular/epididymal inflammation as well because of the so-called systemic cytokine storm typically presented by COVID-19 patients (Cao, 2020), which could trigger further local inflammation and oxidative stress. Interestingly, semen oxidative stress, i.e., an imbalance between reactive oxygen species (ROS) production and antioxidants, has been reported in COVID-19 (Falahieh et al., 2021; Shcherbitskaia et al., 2022). Moreover, increased levels of oxidative stress-induced sperm DNA damage have been reported in COVID-19 patients, with the sperm DNA fragmentation index (DFI) positively correlating with the disease (Falahieh et al., 2021; Gharagozloo et al., 2022). It is well-known that oxidative stress is an important driver of male infertility (Hussain et al., 2023). Oxidative stress and inflammatory cytokines, particularly TNF and IL1β, are known to be detrimental to spermatogonial stem cells and spermatozoa by triggering dysregulated sperm ROS production and inducing apoptosis (Motrich et al., 2006; Guazzone et al., 2009; Paira et al., 2022). In addition, reported data indicate that SARS-CoV-2 infection would inhibit spermatogenesis by inducing testicular cell senescence through MAPK signaling pathway, which is mainly triggered by stress responses like inflammation and oxidative stress (Wang et al., 2022). Moreover, recently reported data show that death of undifferentiated spermatogonia is caused by inflammatory mediators released during the infection rather than by the infection itself (Giannakopoulos et al., 2023). On the other hand, exacerbated cytokine and chemokine production may also trigger an autoimmune reaction with pathological consequences on testicular tissue such as inflammation-induced spermatogenesis impairment and anti-sperm autoantibodies (Xu et al., 2021; Xie et al., 2022). However, and supporting previously reported data (Donders et al., 2022; Paoli et al., 2023), our results showed that COVID-19 was not significantly associated to anti-sperm IgG or IgA autoantibodies.

Other possible infection effects underlying the observed sperm alterations are hormonal imbalances affecting sperm production such as alterations in the hypothalamic-pituitary-gonadal axis. In fact, hypogonadism revealed by low serum levels of LH and testosterone was initially described in hospitalized acutely ill COVID-19 patients (Salonia et al., 2021). However, a systematic meta-analysis of pre-post COVID-19 studies found no significant changes in serum levels of sex-related hormones before and after the infection indicating that hormonal dysregulation is not associated to SARS-CoV-2 infection (Che et al., 2022). Besides, fever is a known factor to alter spermatogenesis and decrease sperm quality (Haghpanah et al., 2021). However, the impact of fever on semen quality of SARS-CoV-2 infected patients have been shown to be negligible (Holtmann et al., 2020; Ballering et al., 2022; Hu et al., 2022). Moreover, mild/asymptomatic COVID-19 has been shown to certainly have detrimental effects on sperm quality, mainly reflected in sperm concentration and motility, which are not related to fever, symptom severity or changes in sex-related hormones (Guo et al., 2021).

In conclusion, our study revealed that patients with mild and/or severe COVID-19 present semen inflammation and reduced sperm quality as revealed by elevated levels of inflammatory cytokines in semen and sperm alterations such as reduced semen volume, lower total sperm counts, and impaired sperm motility and viability. However, all observed sperm alterations returned to baseline values after three or more months post disease. Altogether, our results indicate that COVID-19 associates with semen inflammation and impairs sperm quality early after disease. However, long COVID-19 seems not to include detrimental consequences on male fertility potential since the observed sperm quality alterations reversed to baseline values after 3–6 months after disease recovery, which is equivalent to 1 or 2 spermatogenesis (Weinbauer et al., 2010). These data constitute compelling evidence allowing a better understanding of COVID-19 associated sequelae, fundamental for semen collection in assisted reproduction.

## Data Availability

The raw data supporting the conclusion of this article will be made available by the authors, without undue reservation.
