# Supplementary material for: COVID-19 associates with semen inflammation and sperm quality impairment that reverses in the short term after disease recovery
Source: Front Physiol. 2023 Jul 11;14:1220048. doi: 10.3389/fphys.2023.1220048 (PMC10366368; doi:10.3389/fphys.2023.1220048)
Supplement: Supplementary file 1 [file DataSheet1.PDF]

Supplementary Material

COVID-19 associates with semen inflammation and sperm quality impairment that reverses in the short term after disease recovery

María Sol Martinez[1†], Fernando Nicolás Ferreyra[1†], Daniela Andrea Paira[1], Virginia Elena Rivero[1], José Javier Olmedo[2], Andrea Daniela Tissera[2], Rosa Isabel Molina[2], Rubén Darío Motrich[1*]

[1] CIBICI-CONICET, Facultad de Ciencias Químicas, Universidad Nacional de Córdoba, Argentina.

[2] Fundación Urológica Córdoba para la Docencia e Investigación Médica (FUCDIM), Córdoba, Argentina.

[3] Laboratorio de Andrología y Reproducción (LAR), Córdoba, Argentina.

[†]These authors contributed equally to this work and share first authorship.

*** Correspondence:** Ruben D. Motrich (rmotrich@unc.edu.ar)

Supplementary Table 1: Semen analysis in COVID-19 patients and control individuals

**Semen analysis**	**Subjects (mean ± SD)**					**Lower reference limit** [c]
**Variable**	**Controls** (n=62)	**Mild COVID-19 Patients** (n=199)	***p*** [a]	**Severe COVID-19 Patients** (n=32)	***p*** [b]	
Volume (mL)	3.22 ± 1.46	2.64 ± 1.31	* 0.01	2.43 ± 1.36	* 0.009	≥ 1.5
pH	7.65 ± 0.19	7.65 ± 0.16	> 0.99	7.68 ± 0.18	0.87	≥ 7.2
Sperm concentration (x10^6/mL)	106.10 ± 65.88	108.50 ± 89.70	> 0.99	100.40 ± 92.09	> 0.99	≥ 15.00
Total sperm count (millions)	317.60 ± 196.50	258.70 ± 235.50	* 0.02	236.4 ± 258.50	* 0.03	≥ 39.0
Total sperm motility (%)	52.75 ± 14.39	43.05 ± 20.07	* 0.002	41.44 ± 19.15	* 0.02	≥ 40.0
Progressive sperm motility (%)	34.00 ± 12.96	25.25 ± 15.85	* 0.0001	22.63 ± 14.06	* 0.002	≥ 32
Sperm viability (%)	87.15 ± 5.21	83.93 ± 7.98	* 0.01	86.29 ± 6.30	> 0.99	≥ 58
Peroxidase (+) cells (x10^6/mL)	0.07 ± 0.11	0.30 ± 0.61	0.22	0.23 ± 0.86	0.36	≤ 1.00
Anti-sperm IgG antibodies (MAR test, %)	1.29 ± 1.10	1.73 ± 1.83	0.53	1.27 ± 1.72	> 0.99	< 50
Krüger's morphology (normal sperm, %)	5.98 ± 3.07	5.37 ± 3.12	0.78	5.93 ± 3.44	> 0.99	≥ 4
WHO's morphology (normal sperm, %)	5.98 ± 3.07	5.27 ± 3.16	0.38	5.79 ± 3.49	> 0.99	≥ 4

([a]) Mild COVID-19 patients vs. controls. ([b]) Severe COVID-19 patients vs. controls. ([c]) Lower reference value according to the World Health Organization Semen Analysis Manual 5th Ed. 2010. ND: not determined. MAR: mixed antiglobulin reaction. A * *p*<0.05 was considered statistically significant.

Supplementary Table 2: Follow up semen analysis in COVID-19 patients before and after disease recovery

**Semen analysis**	**Patients (mean ± SD)**					**Lower reference limit** [c]
**Variable**	**Pre COVID-19**	**Early after COVID-19**	***p*** [a]	**Late after COVID-19**	***p*** [b]	
Volume (mL)	2.61 ± 1.24	2.61 ± 1.39	> 0.99	2.45 ± 1.24	> 0.99	≥ 1.5
pH	7.61 ± 0.13	7.61 ± 0.11	> 0.99	7.63 ± 0.14	0.66	≥ 7.2
Sperm concentration ($x10^6$/mL)	98.42 ± 79.06	85.44 ± 79.34	0.97	109.80 ± 74.64	0.55	≥ 15.00
Total sperm count (millions)	255.80 ± 171.04	171.09 ± 152.60	* 0.02	223.00 ± 151.00	0.86	≥ 39.0
Total sperm motility (%)	44.68 ± 20.72	32.70 ± 21.52	* 0.013	43.82 ± 20.27	> 0.99	≥ 40.0
Progressive sperm motility (%)	27.71 ± 16.50	18.36 ± 15.67	* 0.011	26.46 ± 15.89	> 0.99	≥ 32
Sperm viability (%)	83.88 ± 8.29	78.13 ± 11.49	* 0.02	84.95 ± 8.92	0.67	≥ 58
Peroxidase (+) cells ($x10^6$/mL)	0.18 ± 0.29	0.50 ± 1.48	> 0.99	0.32 ± 0.54	> 0.99	≤ 1.00
Anti-sperm IgG antibodies (MAR test, %)	1.76 ± 1.53	1.79 ± 1.97	> 0.99	1.51 ± 1.14	> 0.99	< 50
Krüger's morphology (normal sperm, %)	6.09 ± 3.56	5.18 ± 3.12	0.61	5.79 ± 3.28	> 0.99	≥ 4
WHO's morphology (normal sperm, %)	6.09 ± 3.56	4.84 ± 3.18	0.22	5.64 ± 3.37	> 0.99	≥ 4

([a]) Early after COVID-19 vs. Pre COVID-19. ([b]) Late after COVID-19 vs. Pre COVID-19. ([c]) Lower reference value according to the World Health Organization Semen Analysis Manual 5th Ed. 2010. ND: not determined. MAR: mixed antiglobulin reaction. Patients, n=231. A * p<0.05 was considered statistically significant.
